# Transcriptome analysis of *Artemisia argyi* following methyl jasmonate (MeJA) treatment and the mining of genes related to the stress resistance pathway

**DOI:** 10.3389/fgene.2023.1279850

**Published:** 2023-11-02

**Authors:** Jing Wang, Yupeng Cui, Shuyan Li, Xinqiang Gao, Kunpeng Zhang, Xiangling Shen

**Affiliations:** ^1^ Biotechnology Research Center, China Three Gorges University, Yichang, China; ^2^ College of Biology and Food Engineering, Anyang Institute of Technology, Anyang, China

**Keywords:** *Artemisia argyi*, MeJA, transcriptome, abiotic stress, response

## Abstract

*Artemisia argyi* Lev. et Vant. (*A. argyi*) is a perennial grass in the *Artemisia* family, the plant has a strong aroma. Methyl jasmonate (MeJA) is critical to plant growth and development, stress response, and secondary metabolic processes. The experimental material *Artemisia argyi* was utilized in this study to investigate the treatment of *A. argyi* with exogenous MeJA at concentrations of 100 and 200 μmol/L for durations of 9 and 24 h respectively. Transcriptome sequencing was conducted using the Illumina HiSeq platform to identify stress resistance-related candidate genes. Finally, a total of 102.43 Gb of data were obtained and 162,272 unigenes were identified. Differential analysis before and after MeJA treatment resulted in the screening of 20,776 differentially expressed genes. The GO classification revealed that the annotated unigenes were categorized into three distinct groups: cellular component, molecular function, and biological process. Notably, binding, metabolic process, and cellular process emerged as the most prevalent categories among them. The results of KEGG pathway statistical analysis revealed that plant hormone signal transduction, MAPK signaling pathway-plant, and plant-pathogen interaction were significant transduction pathways in *A. argyi*’s response to exogenous MeJA-induced abiotic stress. With the alteration of exogenous MeJA concentration and duration, a significant upregulation was observed in the expression levels of calmodulin CaM4 (ID: *EVM0136224*) involved in MAPK signaling pathway-plant and auxin response factor ARF (ID: *EVM0055178*) associated with plant-pathogen interaction. The findings of this study establish a solid theoretical foundation for the future development of highly resistant varieties of *A. argyi*.

## 1 Introduction

*Artemisia argyi* lev. et Vant. (*A. argyi*) is a perennial herb widely distributed in temperate, cold temperate and subtropical regions of Asia, Europe and North America. In China, it is mainly distributed in the northeast, north, east, south and southwest provinces (Bora and Sharma, 2011). Because its whole grass can be used as medicine, it has the effects of reducing fever, altering haemostasis, warming meridians, preventing chills, bacteriostasis and anti-inflammation (Wen et al., 2023). Li Yanwen, the father of Li Shizhen, wrote that “*A. argyi* produced in Shanyin, collected in Duanwu, cured diseases and moxibustion, and its function was not small.” Existing studies have found that the main components of *A. argyi* included essential oils, flavonoids, eudesmane and triterpenes, and activities research showed that flavonoids have antioxidant, radicals clearing, anticancer and bacteriostatic activitie (Yoshikawa et al., 1996; Abad et al., 2012; Han et al., 2017). This is why people have begun to pay attention to the chemical composition of mugwort leaf and are committed to improving its useful value.

Methyl jasmonate (MeJA) was originally isolated from the essential oil of Jasminum grandiflorum and is a class of plant stress hormones similar to the salicylates (Cohen and Flescher, 2009). The main relevant substances are JA and MeJA. As a plant stress hormone, MeJA is critical to plant growth and development, stress response, and secondary metabolic processes (Zang et al., 2015). Hu et al. (2013) found that endogenous JA positively regulates the plant response to low temperature stress, the application of exogenous JA enhances plant cold resistance, and inhibiting the biosynthesis and signal transduction of endogenous JA makes plants sensitive to low temperature stress. JA can also improve the ability of plants to cope with biological stress. Yan et al. (2021) found that spraying appropriate concentrations of JAs on leaves significantly reduced the toxic effects of pests on leaves. After JA treatment, the contents of tannins and total phenols in leaves increased significantly. Then, the activity of detoxification enzymes in pests was analysed. It was found that the activity of detoxification enzymes in adults was inhibited to varying degrees, which significantly reduced the leaf feeding area of adults. Muhammad et al. (2021) found that exogenous spraying of JA could enhance the activity of antioxidant enzymes, ascorbic acid, glutathione pools, and the glyoxal enzyme system, induce the initiation of the plant defence system, and alleviate the effects of chromium on photosynthetic pigments, the photosynthetic system and the growth of chrysanthemum. Compared with chromium stress treatment, JA treatment restored the mineral nutrients of chrysanthemum to control levels, limited the absorption of chromium and the accumulation in roots and shoots, and restored the plant growth and physiological and biochemical indexes to the control level. In addition, many studies have shown that jasmonates can have a variety of morphological or physiological effects, such as inhibiting plant growth, inhibiting pollen germination, promoting leaf senescence, promoting abscission, inducing tuber formation, inducing tendril curling, and promoting stomatal closure (Sembdner and Parthier, 1993). At the same time, MeJA is involved in various activities of plants as it acts as a signal substance and plays an important role in various physiological and biochemical processes, such as plant growth and development, metabolism, stress response (such as wound and defence response) and stress response (Zhai et al., 2015; Takaoka et al., 2018; Wasternack and Hause, 2019). Cho et al. (2008) found that exogenous MeJA could act as a signal transduction molecule: it mediated the production of secondary metabolites such as phenols and terpenoids, thereby boosting contents of natural plant chemicals. Therefore, exogenous application of MeJA provides an effective way to explore the key enzyme genes in the process of plant stress and elucidate the related pathways, and can further regulate the production of secondary metabolites in plants to resist external stress.

At present, there are few reports on the resistance of *A. argyi* to exogenous MeJA treatment. Therefore, in this study, *A. argyi* was used as the material to carry out transcriptome research after exogenous MeJA treatment of *A. argyi* and to explore the differentially expressed genes (DEGs) in stress-related pathways to provide a reference for the further study of stress-resistant varieties of *A. argyi*.

## 2 Materials and methods

### 2.1 Plant materials and RNA extraction

The *A. argyi* plants were taken from tissue cultures and brought into a greenhouse, where they were inserted into nutrient medium (nutrient soil: vermiculite = 1:1) for conventional management at 24°C ± 2°C under 16 h light/8 h dark condition. After 2 months, a total of 60 healthy and consistent *A. argyi* plants were selected and divided into five groups with 12 plants in each group. The five groups were placed in a closed chamber (1.2 m × 1.2 m × 0.8 m) separated by plastic film in the plant culture room. Group 1: plants were sprayed with sterile water, and the total spraying volume was 150 mL; Group 2 and Group 3: plants were spraying with 100 μmol/L MeJA solution, and the total spraying volume was 150 mL; Group 4 and Group 5: plants were spraying with 200 μmol/L MeJA solution, and the total spraying volume was 150 mL. After the treatments, the five groups were placed in the plant culture room for routine management. The plant leaves were treated with 100 μmol/L (ES, EB) and 200 μmol/L (YB) MeJA solution for 9 h (ES-9 h, YB-9 h) and 24 h (EB-24 h, YB-24 h), respectively, and sterile water treatment was used as a control (CK). After sampling, the samples were frozen in liquid nitrogen at −80°C for later use. Leaf total RNA samples from the five groups were extracted using Trizol reagent (Invitrogen, United States) according to the manufacturer’s manual. The quality and integrity of the total RNA were evaluated using 0.8% agarose gel electrophoresis and a 2100 Bioanalyzer RNA Nano chip device (Agilent, Santa Clara, CA, United States), respectively. The concentrations were measured using a ND-1000 spectrophotometer (NanoDrop, Thermo Scientific, DE, United States). Only the RNA samples in line with the quality standard (OD260/OD280 ≥ 2.0) were treated with DNase I and then sent to the Biomarker Technologies (BMKGENE, Qingdao, China) for mRNA purification, cDNA library construction and sequencing using the Illumina technology.

### 2.2 Construction of the cDNA library and transcriptome sequencing

The mRNA was enriched by magnetic beads with oligo (dT), and the mRNA was randomly interrupted by fragmentation buffer. Using it as a template, the first cDNA strand was synthesized with six base random primers (Random Hexamers), and then the buffer, dNTPs, RNase H and DNA polymerase I were added to synthesize the second cDNA strand; the cDNA was purified by AMPure XP beads. The purified double-stranded cDNA was subjected to terminal repair, an A-tail was added, the sequencing adapter was connected, and then the fragment size was selected with AMPure XP beads. Finally, the cDNA library was obtained by PCR enrichment. The effective concentration of the library (the effective concentration of the library >2 nM) was accurately quantified by Q-PCR.

### 2.3 Sequencing quality control

Sequencing was performed using the Illumina platform. Reads containing joints were removed; low-quality reads (including reads with a removal ratio of N greater than 10% and reads with a removal mass value of Q ≤ 10 accounting for more than 50% of the entire read) were removed to ensure that reads had high enough quality to ensure the accuracy of the subsequent analysis.

### 2.4 Gene expression quantification and DEGs

The number of mapped reads and the length of transcripts in the samples were normalized. StringTie uses fragments per kilobase of transcript per million fragments mapped (FPKM) (Florea et al., 2013) as a measure of transcript or gene expression level through the maximum flow algorithm. Subsequently, DESeq2 (Love et al., 2014) was used to obtain differentially expressed gene sets between the two biological conditions.

### 2.5 DEG functional annotation and metabolic pathway enrichment analysis

The unigene sequences were compared with the COG, GO, KEGG, KOG, Pfam, Swiss-Prot, eggNOG and NR databases, and the DEGs were classified by GO. The pathway enrichment analysis results of unigenes in KEGG were obtained by R/clusterProfiler.

### 2.6 Quantitative RT-PCR (qRT-PCR) validation

To verify the RNA-Seq results, we performed qRT-PCR analysis for the expression of 12 randomly selected genes using the same RNA samples as the transcriptome sequencing. The total RNA of all samples was isolated by Trizol (Invitrogen, United States) and the first strand cDNA was synthesized from each RNA of about 0.5 µg using the PrimerScript first strand cDNA synthesis kit (TaKaRa, Dalian, China). The gene-specific primers were designed, according to the CDSs of the 12 genes, using Primer v5.0 software. The specific primers used are shown in Supplementary Table S1, the expression of DEGs is shown in Supplementary Table S2. qRT-PCR was run using SYBR premix Ex Taq Kit (Takara, Japan) and a Bio-Rad MiniOpticon Real-Time PCR machine (Bio-Rad, United States) using the same cDNA samples as that in the RNA-seq experiment. The qRT-PCR reaction system with the total volume of 25 μL contained 12.5 μL of SYBR MIX, 0.5 μL of upstream primers, 0.5 μL of downstream primers, 0.5 ng of cDNA template, and the amplification program was as follows: pre-denaturation at 94°C for 30 s, followed by 40 cycles of denaturation at 94°C for 5 s, renaturation at 59°C for 15 s, and extension at 72°C for 10 s. All the data were normalized using *AaActin* gene as reference, and the gene expression level was calculated using 2^−ΔΔCT^ (Livak and Schmittgen, 2001). Three biological repeats and three technical repeats were performed for each selected gene.

## 3 Results and analysis

### 3.1 Sample RNA quality detection and sequencing statistics and quality assessment

The low-quality data (including the proportion of N greater than 10% and the number of bases with mass value Q ≤ 10 accounted for more than 50% of the entire read) and the data containing the adaptor were removed from the raw data obtained by the transcriptome sequencing of 15 samples, and 102.43 Gb of clean data were obtained. At least 19,083,320 clean reads were obtained in each library. The GC content of the transcriptome sequence of 15 samples ranged from 42.73% to 44.24%, which was slightly lower than the AT content. The percentage of Q20 bases in each sample was not less than 97.81%, and the percentage of Q30 bases was not less than 93.89% (Table 1). The sequencing quality was good and thus was used for further experimental analysis.

**TABLE 1:** Statistical table of sequencing data.

Samples	Clean reads	Clean bases	GC content	Q20(%)	Q30(%)
CK-1	22,445,839	6,699,919,184	42.84	98.14	94.58
CK-2	19,717,577	5,889,717,418	42.79	98.05	94.31
CK-3	22,875,206	6,837,746,364	42.73	98.11	94.42
ES-9 h-1	22,815,343	6,824,618,066	44.24	97.97	94.27
ES-9 h-2	21,585,067	6,454,624,204	42.90	97.97	94.17
ES-9 h-3	21,134,342	6,319,098,416	42.73	97.81	93.90
EB-24 h-1	23,107,874	6,911,388,880	43.28	98.00	94.31
EB-24 h-2	21,081,176	6,297,704,266	43.21	97.92	94.04
EB-24 h-3	21,570,063	6,447,112,754	43.02	97.90	94.01
YB-9 h-1	26,228,421	7,836,434,332	43.02	98.07	94.40
YB-9 h-2	25,590,524	7,650,641,674	42.89	97.94	94.07
YB-9 h-3	19,083,320	5,707,541,442	42.88	97.88	93.89
YB-24 h-1	27,972,952	8,357,730,950	43.38	97.87	94.06
YB-24 h-2	28,159,266	8,416,454,346	43.48	97.89	94.08
YB-24 h-3	19,325,218	5,777,793,756	43.59	98.03	94.33

### 3.2 Gene function annotation

**FIGURE 1:**
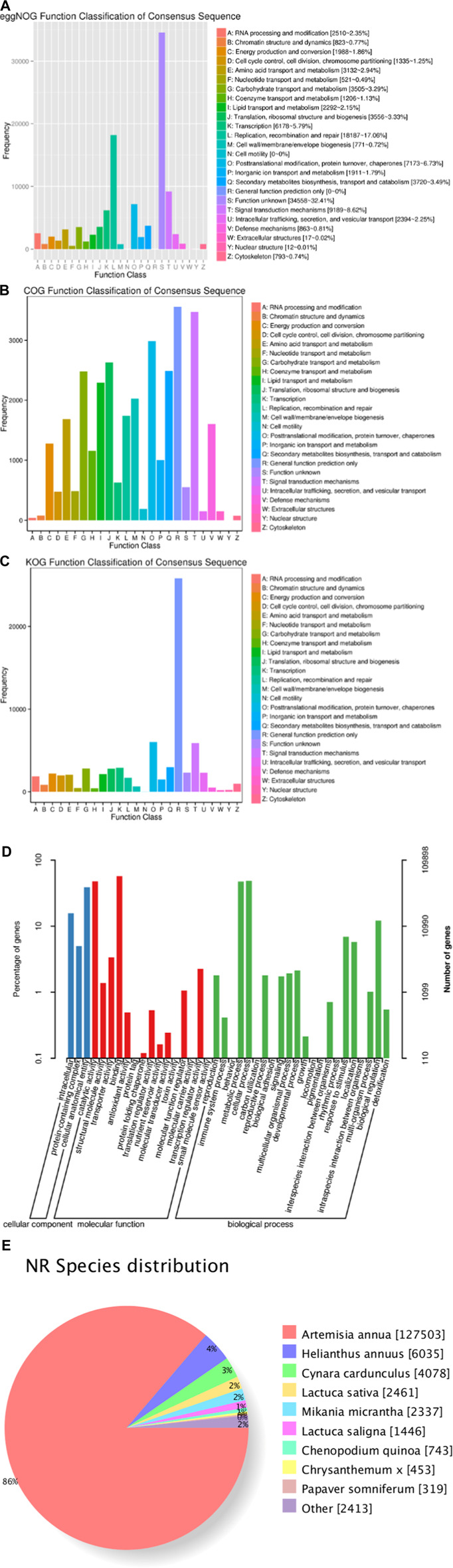
(Continued).

After the transcriptome sequencing of 15 samples, a total of 162,272 unigenes with annotation information were identified. In these databases, the numbers of unigenes annotated to COG, GO, KEGG, KOG, Pfam, Swiss-Prot, eggNOG, and NR were 29,880 (18.4%), 109,903 (67.7%), 87,696 (54%), 65,017 (40.1%), 96,660 (59.6%), 73,528 (45.3%), 106,634 (65.7%), and 147,788 (91.9%), respectively. In addition, a total of 148,296 transcriptome data points were successfully annotated. GO classification results showed that the annotated genes were divided into the following three categories: cellular component, molecular function, and biological process. These three categories of functions were further divided into 40 subcategories. Genes in the cellular component category were mainly concentrated in the cellular anatomical entity (42,770), intracellular (17,194), and protein-containing complex (5,486); the molecular functions were mainly concentrated in binding (62,593), catalytic activity (52,627), and transcription regulator activity (3,698); and the biological processes were mainly focused on cellular process (53,631), metabolic process (51,909), and biological regulation (13,307) (Figure 1; Supplementary Table S3).

### 3.3 Correlation analysis of samples

**FIGURE 2:**
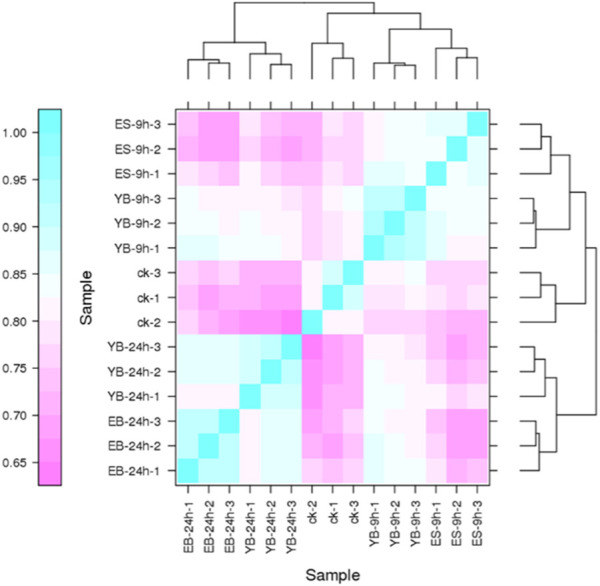
Expression correlation heat map of fifteen samples.

Pearson’s correlation coefficient r is an evaluation index of biological repeated correlation. The closer r^2^ is to 1, the stronger the correlation between the two repeated samples. We ensured that all biological duplicate samples under the same conditions were extracted and constructed with the same batch of samples and sequenced with Run and Lane. It can be seen from the comparison of fifteen samples (Figure 2) that the coefficient between sample repetitions is high, indicating that the data results are reliable.

### 3.4 Identification and functional annotation of DETS

**FIGURE 3:**
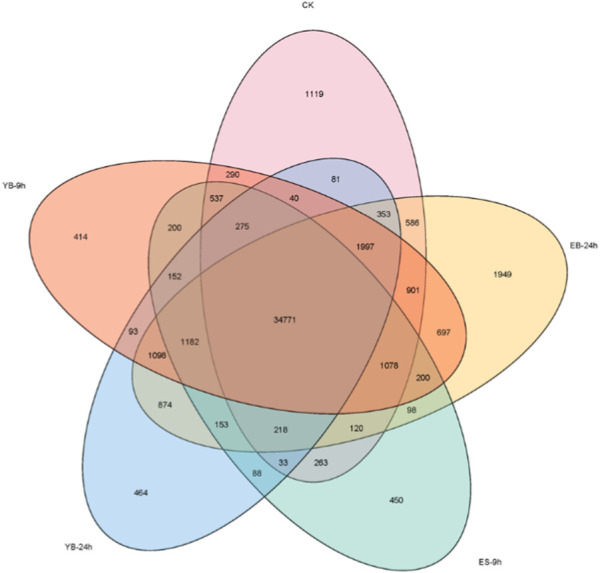
Venn diagram of the quantitative distribution of transcripts (FPKM > 1) expressed in five samples.

To determine the amount of gene expression among the five groups, the FPKM values of each group were calculated to evaluate the transcription level. A total of 162,271 expressed transcripts were identified in each treatment group. With FPKM > 1 as the standard line, 42,662, 39,818, 46,275, 43,925, 41,872 transcripts were detected in CK, ES-9 h, EB-24 h, YB-9 h and YB-24 h, respectively (Figure 3; Supplementary Table S2).

### 3.5 Quantitative analysis of DEGs

**FIGURE 4:**
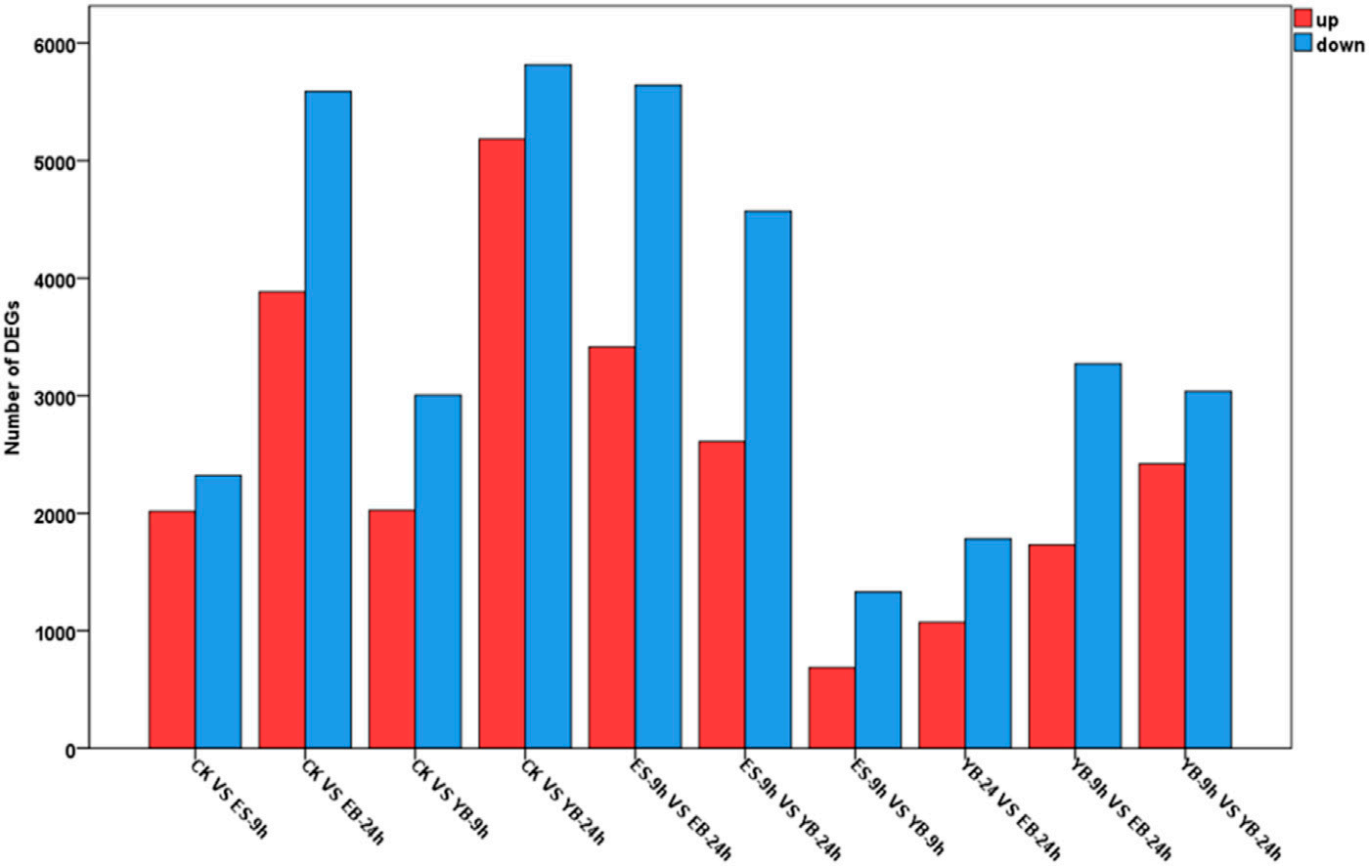
Differential gene expression map.

**FIGURE 5:**
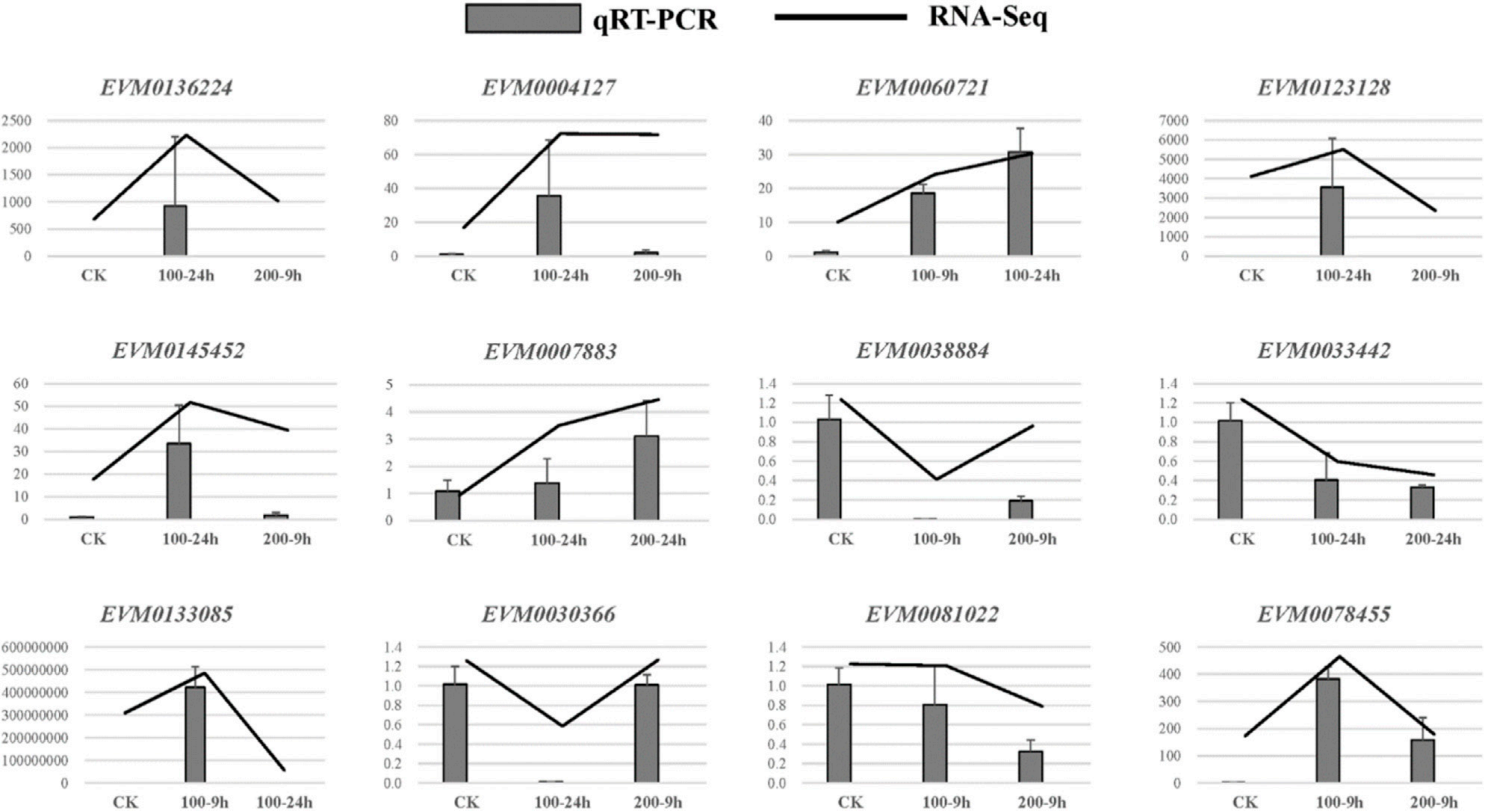
A comparison of the expression profiles of 12 randomly selected DEGs as determined by RNA-Sequencing and qRT-PCR.

In each comparison group, the number of DEGs is shown in Figure 4. Under the control condition, there were 4,340 DEGs between CK and ES-9 h, of which 2,017 were upregulated and 2,323 were downregulated. There were 9,470 DEGs between CK and EB-24 h, of which 3,883 were upregulated and 5,587 were downregulated. There were 5,030 DEGs between CK and YB-9 h, of which 2,026 were upregulated and 3,004 were downregulated. There were 10,999 DEGs between CK and YB-24 h, including 5,183 upregulated genes and 5,816 downregulated genes. There were 9,055 DEGs between ES-9 h and EB-24 h, of which 3,414 were upregulated and 5,641 were downregulated. There were 7,180 DEGs between ES-9 h and YB-24 h, of which 2,609 were upregulated and 4,571 were downregulated. There were 2,015 DEGs between ES-9 h and YB-9 h, of which 685 were upregulated and 1,330 were downregulated. There were 2,853 DEGs between EB-24 h and YB-24 h, including 1,070 upregulated genes and 1,783 downregulated genes. There were 5,002 DEGs between EB-24 h and YB-9 h, of which 1,730 were upregulated and 3,272 were downregulated. There were 10,999 DEGs between YB-9 h and YB-24 h, of which 5,183 were upregulated and 5,816 were downregulated. It can be seen from the data that the number of differentially expressed genes increased with increasing time at the same concentration of *A. argyi.* At the same time, the number of differentially expressed genes increased with increasing concentration (Figure 4; Supplementary Table S4). To validate the quality of our RNA-Seq results, 12 DEGs were randomly selected for their expression analysis by qRT-PCR method. The relative expression levels of these selected genes were strongly consistent with their corresponding FPKM values (Figure 5), thus confirming the validity of the RNA-Seq data.

### 3.6 GO classification and enrichment analysis of DEGs

GO enrichment analysis was performed on all DEGs. In this study, 3,541, 7,729, 4,110, and 8,844 DEGs were annotated in CK vs. ES-9 h, CK vs. EB-24 h, CK vs. YB-9 h, and CK vs. YB-24 h, respectively (Supplementary Figures S1–S4; Supplementary Tables S5–S8).

Among the DEGs in CK vs. ES-9 h, the classification of cellular components mainly focused on membrane (1,133) and membrane part (1,048). Molecular function classification mainly focused on binding (1,922) and catalytic activity (1,742). The classification of biological processes mainly focused on metabolic processes (1,480) and cellular processes (1,388). After treatment with the same concentration, with increasing time, the GO enrichment results were also different. Among the DEGs in CK vs. EB-24 h, the cellular component classification was mainly concentrated on the membrane (2,563) and membrane part (2,335). Molecular function classification mainly focused on catalytic activity (4,046) and binding (4,021). The classification of biological processes mainly focused on metabolic processes (3,501) and cellular processes (3,222).

Among the DEGs in CK vs. YB-9 h, the classification of cellular components mainly focused on membrane (1,332) and membrane part (1,220). The molecular function classification mainly focused on catalytic activity (2,128) and binding (2,060). The classification of biological processes mainly focused on metabolic processes (1,759) and cellular processes (1,561). Among the DEGs in CK vs. YB-24 h, the cellular component classification was mainly concentrated in membrane (3,072) and membrane part (2,809). The molecular function classification mainly focused on catalytic activity (4,463) and binding (4,338). The classification of biological processes mainly focused on metabolic processes (4,033) and cellular processes (3,591).

In our study, GO annotation results showed that *A. argyi* under MeJA treatment could significantly effect the metabolic process; with the passage of time and an increase in concentration, the number of DEGs increased.

### 3.7 KEGG metabolic pathway analysis of DEGs

Through the comparison of the KEGG database, the effects of MeJA treatment on metabolic pathways were reflected. CK vs. ES-9 h, CK vs. EB-24 h, CK vs. YB-9 h, CK vs. YB-24 h each had 1,197, 2,703, 1,396, and 2,951 differential genes mapped to the KEGG pathway, respectively (Supplementary Figures S5–S8; Supplementary Tables S9–S12).

In the CK vs. ES-9 h group, the most involved pathways were plant‒pathogen interactions (246), starch and sucrose metabolism (157), plant hormone signal transduction (131) and MAPK signalling pathway-plants (107).

In the CK vs. EB-24 h group, the most involved pathways were plant‒pathogen interactions (459), plant hormone signal transduction (295) and endocytosis (260).

In the CK vs. YB-9 h group, the most involved pathways were plant‒pathogen interactions (252), starch and sucrose metabolism (173), and protein processing in endoplasmic reticulum (147).

In the CK vs. YB-24 h group, the most involved pathways were plant‒pathogen interaction (520), starch and sucrose metabolism (316), and endocytosis (284).

Further transcriptome analysis showed that plant hormone signal transduction and the MAPK signalling pathway-plant pathway were also related to the response of *A. argyi* to MeJA treatment.

### 3.8 Expression analysis of key genes in stress-related metabolic pathways of *A. argyi*

**FIGURE 6:**
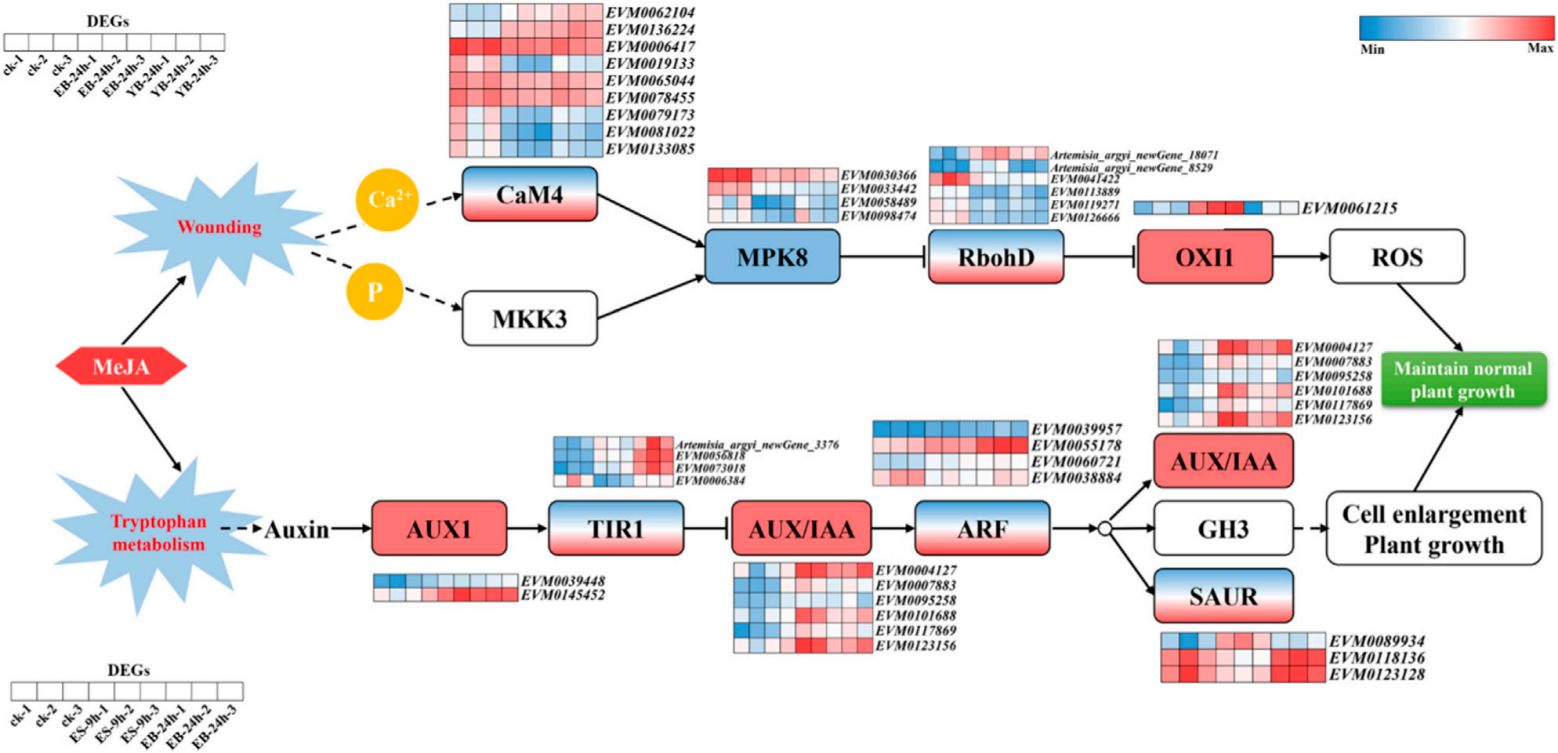
Metabolic pathway of stress-related genes in *A. argyi*.

As shown in Figure 6, *AaOXI1* is an upregulated gene (ID: *EVM0061215*), and *AaMPK8* is a downregulated gene (ID: *EVM0030366*; *EVM0033442*; *EVM0058489*; *EVM0098474*) in the MAPK pathway related to mechanical injury. *AaCaM4* (ID: Up: *EVM0062104*; *EVM0136224*; Down: *EVM0006417; EVM0019133; eVM0065044*; *EVM0078455*; *EVM0079173*; *EVM0081022*; *EVM0133085*) and *AaRbohD* (ID: Up: *Artemisia_argyi_newGene_18071; Artemisia_argyi_newGene_8529*; Down: *EVM0041422*; *EVM0113889*; *EVM0119271*; *EVM0126666*) had genes with both upregulated and downregulated expression. An *AaCaM4* gene was identified, gene ID: *EVM0136224*, which was significantly upregulated in EB-24 h and YB-24 h. The FPKM value was 32.7 in CK, 107.2 in EB-24 h, and 154.0 in YB-24 h.

The changes in the plant hormone signal transduction pathway and related genes are shown in Figure 6. *AaAUX1* (ID: *EVM0039448*; *EVM0145452*) and *AaAUX/IAA* (ID: *EVM0004127*; *EVM0007883*; *EVM0095258*; *EVM0101688*; *EVM0117869*; *EVM0123156*) were upregulated genes; auxin receptor *AaTIR1* (ID: Up: *Artemisia_argyi_newGene _3376*; *EVM0056818*; *EVM0073018*; Down: *EVM0006384*), *AaARF* (ID: Up: *EVM0039957*; *EVM0055178*; *EVM0060721*; Down: *EVM0038884*), and *AaSAUR* (ID: Up: *EVM0089934*; Down: *EVM0118136*; *EVM0123128*) had both upregulated and downregulated genes. Among them, the auxin-related factor *AaARF*, gene ID: *EVM0055178*, was significantly upregulated at ES-9 h and EB-24 h. The FPKM value was 27.7 in CK, 53.9 in ES-9 h and 151.2 in EB-24 h.

## 4 Discussion

*A. argyi* is a plant of Compositae family, which is an important Chinese herbal medicine. However, previous studies mainly focused on the extraction of essential oil and the analysis of aromatic components, and few studies on its functional genomics (Cui et al., 2021; Zhang et al., 2022). In some public databases, including NCBI, only a few complete or partially encoded nucleotide sequences have been published. No EST or Genomic Survey sequence (GSS) of *A. argyi* was found in the GenBank database. Therefore, transcriptome sequencing data of *A. argyi* were analyzed and reported in this study. MeJA are important cell regulators involved in various developmental processes and plant defence and stress responses (Wasternack and Hause, 2013). However, there is little research on the defence and stress response of *A. argyi*. This study is the first to use MeJA treatment, which provides some useful information for further genetic research of this species.

In this study, we obtained 102.43 Gb of clean data by high-throughput transcriptome sequencing. The clean reads obtained by sequencing were 19,083,320∼28,159,266, and the GC content of transcriptome sequences ranged from 42.73% to 44.24%. The percentage of Q20 bases in each sample was not less than 97.81%, and the percentage of Q30 bases was not less than 93.89%. The gene expression trend verified by qRT‒PCR was basically consistent with the FKPM value of the transcriptome. All genes were annotated to COG, GO, KEGG, KOG, Pfam, Swiss-Prot, eggNOG and NR databases. The annotation of these unique genes revealed significant transcriptional complexity and provided information on the expression of valuable genes in *A. argyi* transcriptome, where many DEGs are involved in the biosynthesis and metabolism of plant stress responses. This will provide information for further understanding of the biosynthetic pathways of *A. argyi* in response to MeJA stress.

After exogenous MeJA treatment, a total of 109,903 DEGs in the transcriptome of *A. argyi* were annotated in the GO database. The main enriched GO functions were cell disintegration, intracellular, containing protein complexes, joint sites, catalytic activity, transport activity, cell process, metabolic process, biological regulation. That so many different GO terms were annotated highlights the genes’ diversity likely present in the MeJA treated leaf transcriptomes of *A. argyi* plants. At the same time, a total of 87,696 differential genes were mapped to the KEGG database. After different time and concentration of MeJA treatment, there were differentially expressed genes with significant changes in expression levels among plant hormone signal transduction and the MAPK signalling pathway-plant, the plant‒pathogen interaction, respectively (Figure 6). Those results suggested that they may have played active roles in the regulation of MeJA-responsive transcriptional regulation of the three pathway.

### 4.1 Ca^2+^ signal transduction and ROS elimination in response to exogenous MeJA

When plants are stimulated by the external environment, their homeostasis is disrupted and excessive ROS are produced, which constitutes the early response of plants under various biological or abiotic stress conditions. This can induce thickening of the cell wall, stimulate local allergic death of plant cells, induce the synthesis of plant protection hormones, and activate transcription factors, etc. (Meyer et al., 1993). The rapid production of MeJA increases the accumulation of H_2_O_2_ and Ca^2+^ in leaves, inducing calcium signaling (Rodriguez et al., 2010). CaMs are ubiquitous Ca^2+^ binding proteins that mediate major intercellular Ca^2+^ signaling pathways. Elevated cytoplasmic Ca^2+^ concentrations lead to the formation of active Ca^2+^/CaM complexes, which in turn regulate cell function by interacting with regulatory proteins such as transcription factors, protein kinases and phosphatases as well as ion transporters (Takahashi et al., 2011). In plants, mitogen-activated protein kinase (MAPK) cascades regulate various cellular processes in response to a wide range of biological and abiotic stresses (Rodriguez et al., 2010). The activation of MPK8 requires two pathways: the phosphorylation of MKK3 and Ca^2+^ dependent CaM binding. These two activation modes converging at MPK8 are an important part of monitoring or maintaining ROS homeostasis. *RbohD* is one of the *Rboh* genes and plays a key role in ROS production and signal transduction. The enzyme activity of *RbohD* is regulated by calcium ion binding and protein phosphorylation. In other words, MPK8 negatively regulates the expression of OXI1 by inhibiting the expression of *RbohD* during mechanical damage (Ogasawara et al., 2008), thereby inhibiting the overproduction of ROS. MPK8 plays roles related to connexin phosphorylation, Ca^2+^, and ROS in trauma signaling pathways. MPK8 negatively regulates the expression of RbohD, resulting in the inhibition of ROS production and the subsequent ROS-related signal response after mechanical injury, preventing the excessive accumulation of ROS in cells and ensuring and maintaining appropriate concentrations of ROS, as the proper regulation of ROS is essential for the survival of organisms. We analyzed the gene (ID: *EVM0136224*) encoding *CaM4* and found that its expression was significantly upregulated after treatment at different concentrations for 24 h. When there is an increase in Ca^2+^, MPK8 is activated, the excessive production of ROS is inhibited, and damage to plant cells is reduced, to maintain the normal growth of plants.

### 4.2 Auxin signal transduction pathway in response to exogenous MeJA in *A. argyi*

With the development of biotechnology, studies have found that plant hormones play an important role in regulating the synthesis of secondary metabolites, and the regulatory process involves a variety of signal transduction and interaction factors (Zhao et al., 2005). For example, in Robert-Seilaniantz study, Arabidopsis miRNA393 targets auxin receptors, and its overexpression regulates the accumulation of gluconate in response to defence responses and increases plant resistance to biological nutrition (Robert-Seilaniantz et al., 2011). Ethylene and JA promote the expression of multiple members of AP2/ERF, in which the ORCA3 protein can promote the accumulation of alkaloids, and the regulatory mechanism involves regulating the expression of its synthase (Van der Fits and Memelink, 2001). In response to exogenous MeJA, most genes in the auxin signal transduction pathway were differentially expressed, including *AUX1*, *TIR1*, *AUX/IAA*, *ARF*, and *SAUR*. To date, five auxin biosynthetic pathways have been proposed, including four Trypto-Phan (Trp)-dependent pathways and one tryptophan-independent pathway (Stepanova et al., 2008; Ljung, 2013). With the treatment of exogenous MeJA, tryptophan metabolism will be transformed into auxin, and the auxin signal transduction pathway is composed of auxin receptor TIR1/AFB (Transport Inhibitor Response 1/Auxin Signalling F-Box), transcription inhibitor AUX/IAA, auxin response factor ARF and downstream target genes (Salehin et al., 2015). Auxin can regulate the expression of hundreds of genes, and the main auxin early response genes include the following three major gene families: *AUX/IAA*, *GH3* (*Gretchen Hagen3*) and *SAUR* (*Small Auxin Up RNA*). When the auxin concentration is low, free *AUX/IAA* forms a heterodimer with *ARF* to inhibit the expression of auxin-responsive genes. When the concentration of auxin increases, auxin binds to the *TIR1/AFB* protein, causing *AUX/IAA* ubiquitination and degradation and releasing *ARF*, thereby promoting the expression of auxin-responsive genes (Dharmasiri et al., 2005; Kepinski and Leyser, 2005; Tan et al., 2007). The TIR1/AFB-AUX/IAA-ARF signalling pathway clearly explains the process of auxin perception, conduction and response. Among them, the *ARF* gene family is also involved in the plant stress response. The response mechanism of auxin in plants is very complex. As an important factor in the auxin signal transduction pathway, the *ARF* gene family plays an important role in further analysing the regulatory mechanisms underlying plant growth and development. We found that the gene (ID: *EVM0055178*) in the *ARF* gene family was significantly upregulated at both concentrations after 9 h of treatment at different concentrations, thereby promoting the rapid expression of corresponding genes, promoting cell growth, and helping maintain the normal growth of plants.

## 5 Conclusion

In this study, a total of 20,776 differential genes were screened out through transcriptome sequencing analysis of *A. argyi* leaves sprayed with exogenous MeJA. The GO classification showed that binding, metabolic process and cellular process were the most annotated genes. Statistical analysis of KEGG pathway showed that Plant hormone signal transduction, MAPK signaling pathway-plant, Plant-pathogen interaction are important transduction pathways of *A. arygi* in response to exogenous MeJA stress. We screened out two significantly upregulated genes *CaM4* and *ARF* in Plant hormone signal transduction and MAPK signaling pathway-plant pathway respectively under different contrast conditions, and proposed models for them. These results provide a theoretical basis for further research on the molecular mechanism of plant hormone signal transduction and MAPK signal pathway, and how to improve resistance of *A. argyi* leaf through gene regulation and genetic engineering.

## Data Availability

The original contributions presented in the study are publicly available. The final assembly and FPKM data were submitted to Figshare (https://figshare.com/) under the DOI 10.6084/m9.Figshare.24159120.
