# Supplementary material for: Transcriptome analysis of Artemisia argyi following methyl jasmonate (MeJA) treatment and the mining of genes related to the stress resistance pathway
Source: Front Genet. 2023 Nov 2;14:1279850. doi: 10.3389/fgene.2023.1279850 (PMC10652873; doi:10.3389/fgene.2023.1279850)
Supplement: Supplementary file 5 [file DataSheet1.PDF]

Supplementary

1 Supplementary Figures and Tables

1.1 Supplementary Figures

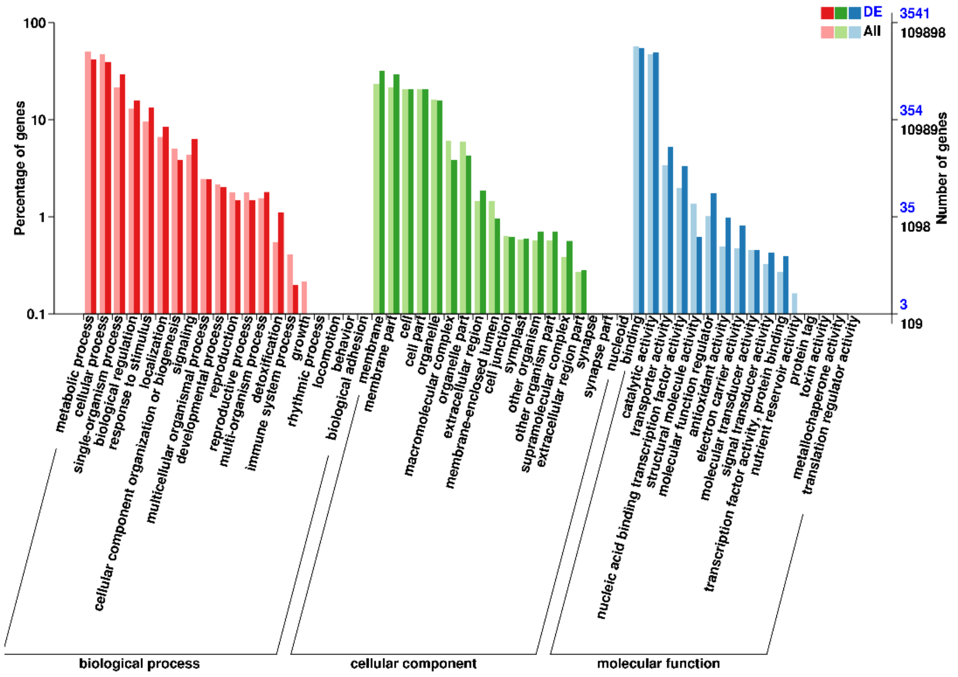

Supplementary Figure 1. GO Classification and Enrichment Analysis of DEGs: CK vs ES-9h

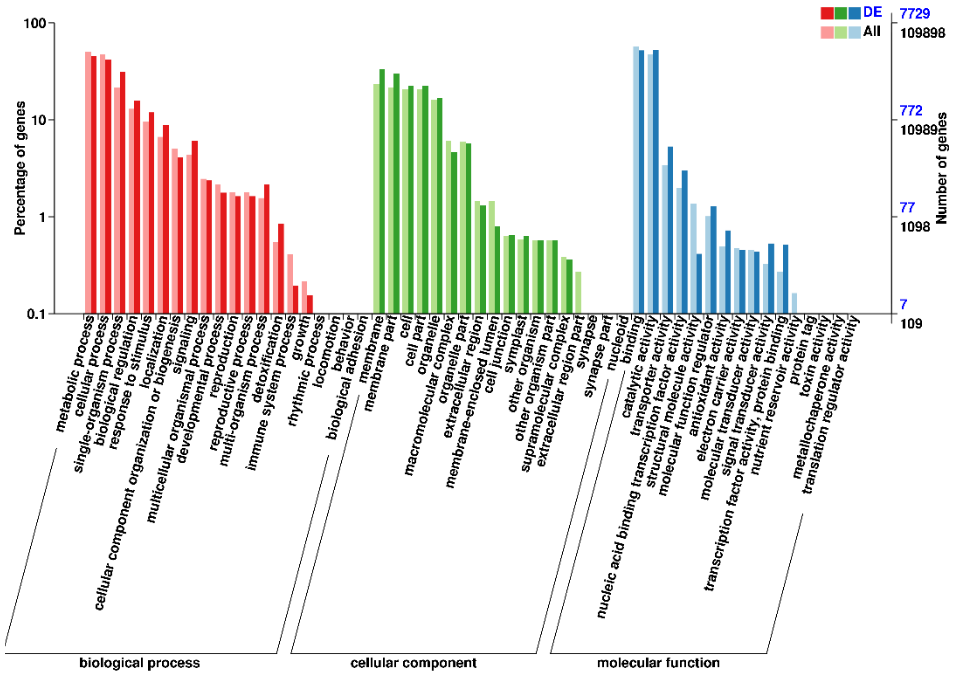

Supplementary Figure 2. GO Classification and Enrichment Analysis of DEGs: CK vs ES-24h

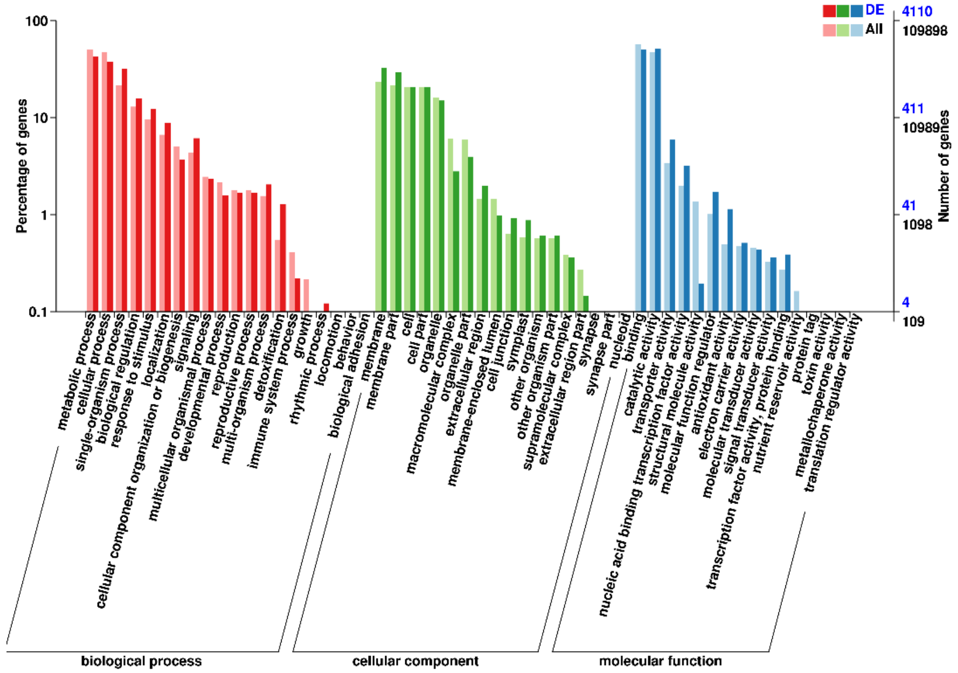

Supplementary Figure 3. GO Classification and Enrichment Analysis of DEGs: CK vs YB-9h

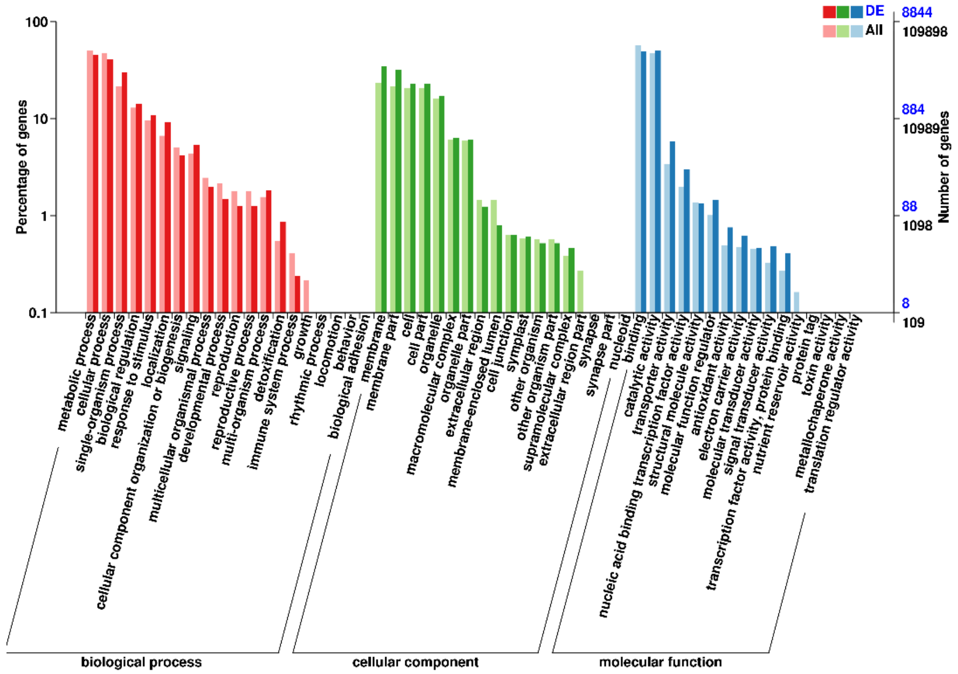

Supplementary Figure 4. GO Classification and Enrichment Analysis of DEGs: CK vs YB-24h

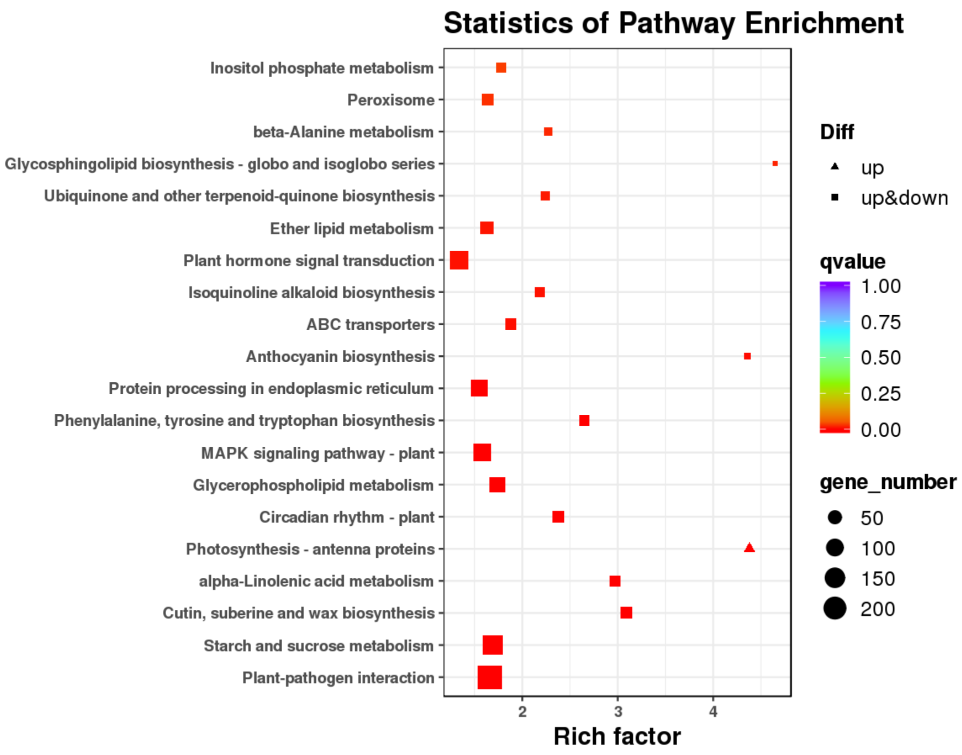

Supplementary Figure 5. KEGG Metabolic Pathway Analysis of DEGs: CK vs ES-9h

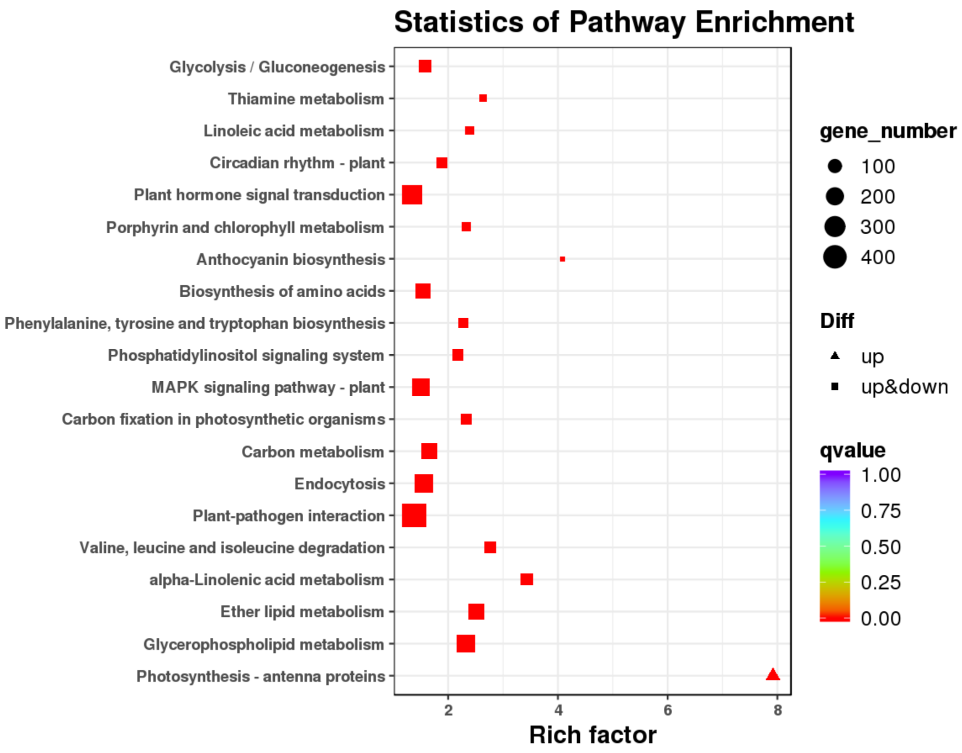

Supplementary Figure 6. KEGG Metabolic Pathway Analysis of DEGs: CK vs EB-24h

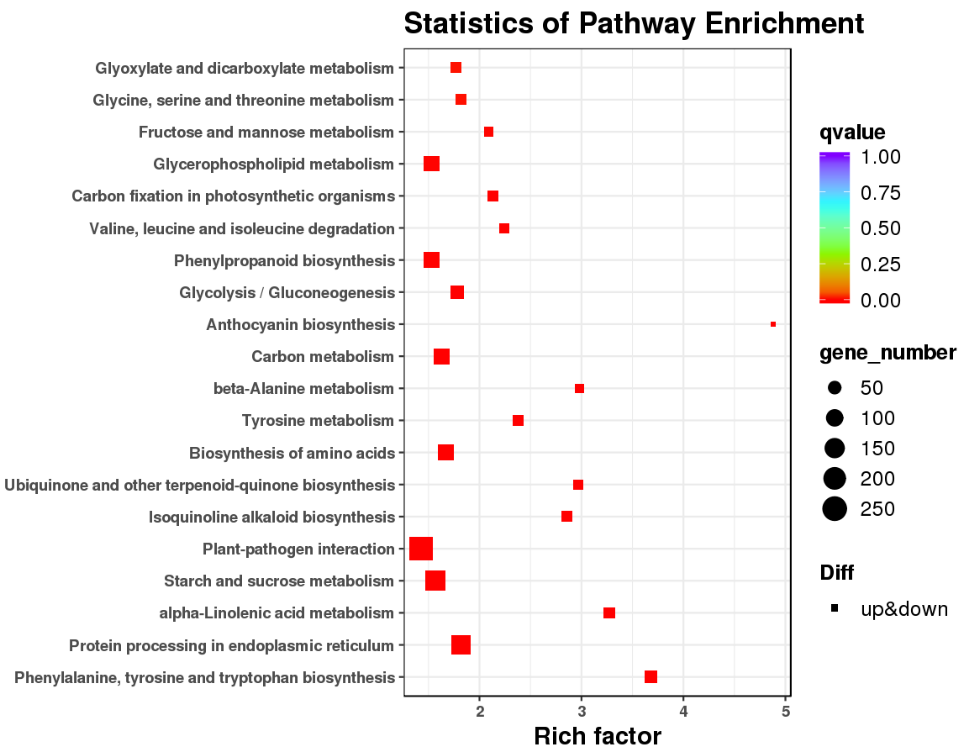

Supplementary Figure 7. KEGG Metabolic Pathway Analysis of DEGs: CK vs YB-9h

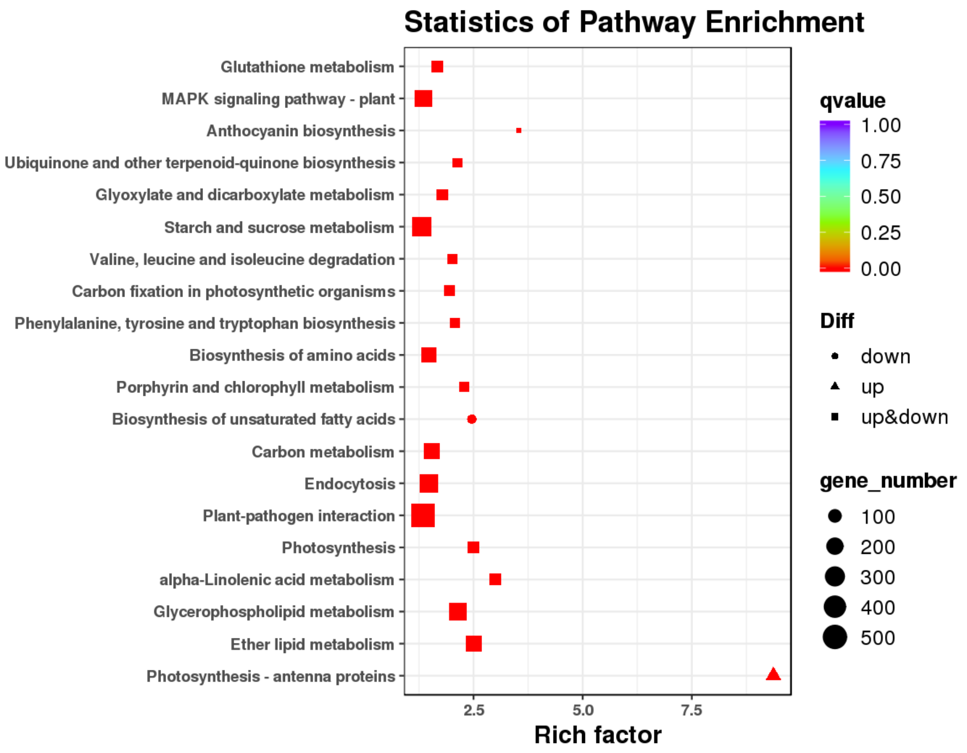

Supplementary Figure 8. KEGG Metabolic Pathway Analysis of DEGs: CK vs YB-24h
