# Supplementary material for: Transcriptome analysis of Artemisia argyi following methyl jasmonate (MeJA) treatment and the mining of genes related to the stress resistance pathway
Source: Front Genet. 2023 Nov 2;14:1279850. doi: 10.3389/fgene.2023.1279850 (PMC10652873; doi:10.3389/fgene.2023.1279850)
Supplement: Supplementary file 6 [file Table5.DOCX]

**Supplementary Table 3 Number of differentially expressed genes**

| **DEG Set** | **DEG Number** | **up-regulated** | **down-regulated** |
| --- | --- | --- | --- |
| ck-1_ck-2_ck-3_vs_ES-9h-1_ES-9h-2_ES-9h-3 | 4,340 | 2,017 | 2,323 |
| ck-1_ck-2_ck-3_vs_EB-24h-1_EB-24h-2_EB-24h-3 | 9,470 | 3,883 | 5,587 |
| ck-1_ck-2_ck-3_vs_YB-9h-1_YB-9h-2_YB-9h-3 | 5,030 | 2,026 | 3,004 |
| ck-1_ck-2_ck-3_vs_YB-24h-1_YB-24h-2_YB-24h-3 | 10,999 | 5,183 | 5,816 |
| ES-9h-1_ES-9h-2_ES-9h-3_vs_EB-24h-1_EB-24h-2_EB-24h-3 | 9,055 | 3,414 | 5,641 |
| ES-9h-1_ES-9h-2_ES-9h-3_vs_YB-24h-1_YB-24h-2_YB-24h-3 | 7,180 | 2,609 | 4,571 |
| ES-9h-1_ES-9h-2_ES-9h-3_vs_YB-9h-1_YB-9h-2_YB-9h-3 | 2,015 | 685 | 1,330 |
| YB-24h-1_YB-24h-2_YB-24h-3_vs_EB-24h-1_EB-24h-2_EB-24h-3 | 2,853 | 1,070 | 1,783 |
| YB-9h-1_YB-9h-2_YB-9h-3_vs_EB-24h-1_EB-24h-2_EB-24h-3 | 5,002 | 1,730 | 3,272 |
| YB-9h-1_YB-9h-2_YB-9h-3_vs_YB-24h-1_YB-24h-2_YB-24h-3 | 5,458 | 2,421 | 3,037 |
