# Supplementary material for: Transcriptome analysis of Artemisia argyi following methyl jasmonate (MeJA) treatment and the mining of genes related to the stress resistance pathway
Source: Front Genet. 2023 Nov 2;14:1279850. doi: 10.3389/fgene.2023.1279850 (PMC10652873; doi:10.3389/fgene.2023.1279850)
Supplement: Supplementary file 8 [file Table4.DOCX]

**Supplementary Table 1 Gene function annotation results**

| **Annotated databases** | **Gene Number** | **Annotated_Percent** |
| --- | --- | --- |
| COG | 29,880 | 18.4 |
| GO | 109,903 | 67.7 |
| KEGG | 87,696 | 54 |
| KOG | 65,017 | 40.1 |
| Pfam | 96,660 | 59.6 |
| Swiss-Prot | 73,528 | 45.3 |
| eggNOG | 106,634 | 65.7 |
| Nr | 147,788 | 91.1 |
| All | 148,296 | 91.4 |
